# An Assessment of Melatonin Levels in the Saliva of Patients with Chronic Urticaria in Comparison with Their Sleep Quality and Dermatologic Quality of Life

**DOI:** 10.3390/clinpract16020036

**Published:** 2026-02-06

**Authors:** Iva Bešlić, Alen Vrtarić, Ivana Škrinjar, Ema Barac, Ana-Karla Vodanović, Liborija Lugović-Mihić

**Affiliations:** 1 School of Dental Medicine, University of Zagreb, Gundulićeva 5, 10000 Zagreb, Croatia; ivaaabukvic@gmail.com (I.B.); iskrinjar@sfzg.hr (I.Š.); 2 Department of Clinical Chemistry, University Clinical Hospital Center Sestre Milosrdnice, Vinogradska 29, 10000 Zagreb, Croatia; alenvrtaric@gmail.com; 3 Department of Oral Medicine, University Hospital Centre Zagreb, Ulica Mije Kišpatića 12, 10000 Zagreb, Croatia; 4 Family Physician Office, Švarcova 20, 10000 Zagreb, Croatia; ema.barac@gmail.com; 5 Association for Scientific and Educational for Highschoolers, Kvaternikova ulica 92a, 10000 Zagreb, Croatia; anakarla.vodanovic@gmail.com; 6 Department of Dermatovenereology, University Hospital Center Sestre Milosrdnice, Vinogradska 29, 10000 Zagreb, Croatia

**Keywords:** urticaria, chronic spontaneous urticaria, melatonin, urticaria activity score, salivary diagnostics, itching, sleep disorders, psychological disorders, quality of life, urticaria control test

## Abstract

**Background:** For the majority of chronic spontaneous urticaria (CSU) sufferers, nocturnal itch has a profound effect on quality of life (QoL), as it leads to sleep disturbances. To ensure good sleep quality (SQ), the body must produce an adequate amount of melatonin, which regulates the sleep cycle. **Methods:** This study examines the levels of salivary melatonin in 38 CSU patients and 38 healthy controls, as well as the relationship between CSU severity, QoL and SQ. The Enzyme-Linked Immunosorbent Assay (ELISA), Dermatology Quality of Life Index (DLQI), and Pittsburgh Sleep Quality Index (PSQI) were used to determine salivary melatonin levels, QoL, and SQ. In addition, the CSU participants were given the Urticaria Activity Score (UAS) and the Urticaria Control Test. **Results:** The median value of salivary melatonin in CSU patients was lower than that in healthy individuals (0.2 vs. 15.985 pg/mL; *p* < 0.001). A decreased melatonin level was seen in 90% of CSU patients and 18% of healthy individuals. Individuals with lower melatonin levels were significantly more likely to have CSU compared with those with higher melatonin levels (OR = 37.6; 95% CI 10.0–141.1). Melatonin was linearly related to QoL and sleep quality in the whole sample (*r* = −0.606 and −0.536; *p* < 0.001) but not in CSU patients. Impaired QoL in patients correlated with itch intensity and the number of hives (*r* = 0.740 and 0.646). The severity and activity of CSU are linearly related to impaired QoL and sleep quality (r = −0.606 and −0.536; *p* < 0.001). Sleep quality acts as the mediator of the association between QoL and salivary melatonin, when controlling for the effect of age and gender (B = −0.347; 95% CI = −0.679 to −0.080). **Conclusions:** The data suggest that melatonin may be more a non-specific marker of sleep disturbance than the severity of CSU. Sleep quality may act as a mediator linking dermatology-related QoL, circadian dysregulation and reduced melatonin secretion.

## 1. Introduction

Chronic spontaneous urticaria (CSU) is characterized by the appearance of hives often accompanied by angioedema, lasting longer than six weeks, with an intense itching sensation and often consequently poor quality of life (QoL) [1,2,3,4]. In CSU patients, sleep disorders and psychological disorders (depression, anxiety) are common. Disturbed sleep primarily involves difficulties in falling asleep, early awakening and a feeling of fatigue, which often negatively impact the individual’s professional and social life. In CSU pathogenesis, various factors may be involved and may trigger pathogenetic pathways, including autoimmune processes and autoantibodies (e.g., IgG, Toll-like receptors, neuropeptides, cytokines), infections, allergens, physical factors, stress, intestinal dysbiosis, vitamin D deficiency, and other triggers which, through various molecular pathways, may lead to mast cell degranulation [4]. In CSU, key pathogenetic events are mast cell and basophil activation and degranulation, which are mediated by autoantibodies. The skin infiltration of mast cells, basophils, eosinophils, T cells, monocytes, and neutrophils indicates a late-phase cutaneous reaction following allergen exposure, with a cytokine profile predominantly reflecting a Th2 response. This infiltration also includes components of Th1 and Th17 inflammation, with their associated immune (e.g., cytokines) and endocrine factors.

When considering many factors related to a person’s sleep (which are not always possible to measure), it is vital to highlight the role of melatonin, N-acetyl-5-methoxytryptamine, a hormone very important for the sleep cycle [5,6]. It initiates and maintains the sleep cycle, which is the basis for using melatonin as a therapeutic option for specific conditions. The process of melatonin synthesis begins with the amino acid tryptophan. Hypothalamic suprachiasmatic paraventricular nuclei control the synthesis of melatonin in the pinealocytes of the pineal gland, and according to the circadian rhythm, melatonin is produced daily in synchronization with the light–dark cycle. Melatonin is a lipophilic molecule, and during the night, it easily diffuses into the cerebrospinal fluid and the bloodstream (where it is mainly bound to albumin, about 70% of total melatonin) [7,8].

Melatonin is a key player in circadian rhythms and an inducer of antioxidant defense, while some other properties still require independent confirmation (immune responses, apoptosis, or mitochondrial homeostasis) [5,6,9,10]. For the measurement of melatonin, the values of 6-sulfatoxymelatonin created in the urine must reflect plasma melatonin levels. In addition to urine, melatonin can also be measured/excreted in saliva (as a free form that is not bound to albumin) [8]. Melatonin values in saliva range from daytime levels (between 1 and 5 pg/mL) to nighttime levels (which range from 10 to 50 pg/mL) [9]. The use of salivary melatonin for diagnostic purposes has many advantages because it is a non-invasive, simple and reliable method for monitoring its rhythm and level, reflecting the free fraction that is active (useful in the diagnosis of sleep disorders, chronic diseases and potentially oral cancer), offering insight into QoL and endogenous circadian rhythm. In addition, salivary melatonin values were found to strongly correlate with their serum levels. Saliva sampling is easy to perform and more comfortable than blood sampling, especially for multiple/frequent measurements and for children. Salivary melatonin reflects the free, biologically active level of the hormone, as opposed to total melatonin in the blood.

According to our recent pilot study results, in the majority of CSU patients, decreased salivary melatonin levels were found (86%), with significant correlation between their lower salivary melatonin values and reduced QoL [6]. Since melatonin levels have not yet been widely studied in CSU, we wanted to determine melatonin concentrations in CSU patients in relation to other clinical indicators of the disease.

## 2. Materials and Methods

### 2.1. Participants

We conducted a case–control, cross-sectional study at the Department of Dermatovenereology and the Department of Clinical Chemistry, Sestre Milosrdnice University Hospital Centre, University of Zagreb, Zagreb, Croatia, in the period between October 2021 and June 2022. This study was approved by the Ethics Committee of the aforementioned hospital (003-06/21-03/030). Each participant received detailed instructions on conducting this study.

This study included two groups of participants: patients with CSU and healthy subjects. Inclusion criteria for the CSU group were a diagnosis of CSU (chronic urticaria of unknown etiology lasting more than six weeks) and age between 18 and 65 years, while we excluded those with chronic induced urticaria, acute urticaria, or urticarial vasculitis; patients with oral pathological conditions (periodontal disease, oral infections or other inflammatory processes, oral ulcers, and cancer, xerostomia); patients with malignant disease or mental disorders; pregnant and breastfeeding women; those working night shifts; and patients on systemic corticosteroids, omalizumab, or other immunosuppressive therapy.

This study included 38 CSU patients who met the mentioned criteria and signed consent forms and 38 healthy subjects (control group), matched by age and sex, who did not suffer from malignant or psychiatric diseases. Each patient was examined by a dermatologist–allergist, followed by an oral pathologist (from the School of Dental Medicine) to exclude pathological conditions in the oral cavity. Sample size calculation was made using the on-line tool Statulator (http://statulator.com/SampleSize/ss2M.html), accessed on 15 February 2020. A minimal sample of 36 from each group was needed to detect a hypothetical difference of 3 units in melatonin between subjects with CSU and healthy controls, with a pooled standard deviation of 4 units, 80% power and a level of significance of 0.05.

### 2.2. Methods

#### 2.2.1. Determining Disease Characteristics Using Standard Questionnaires for Assessing Activity of CSU, Disease Control, DLQI, and Sleep Quality

In CSU patients, four standardized questionnaires were used to collect data on the activity of CSU, disease control, dermatological QoL, and sleep quality. Patients completed four questionnaires: Urticaria Activity Score (UAS7), Urticaria Activity Score (UCT), The Dermatology Life Quality Index (DLQI), and Pittsburgh Sleep Quality Index (PSQI). The DLQI and PSQI were completed exactly on the day of saliva sampling. The control group of healthy subjects only completed the DLQI and PSQI questionnaires (also on the day of saliva sampling). They did not complete questionnaires about urticaria because they do not have urticaria.

For the analysis of urticaria activity, the UAS7 questionnaire (diary-keeping) was used, which considers the number of wheals and the intensity of itching tracked over seven days [1,11,12,13,14]. On a single day, the score can range from 0 to 6, based on the number of wheals (0 = no wheals; 1 = 1–6 wheals; 2 = 7–12 wheals; 3 = more than 12 wheals) and the intensity of itching (0 = no itching; 1 = mild; 2 = moderate; 3 = severe itching), resulting in a seven-day score that can range from 0 to 42. Based on the obtained UAS7 scores, CSU is classified as mild (up to 15), moderate (16–27), or severe (above 28) CSU.

The UCT, a standardized questionnaire, was used to assess the effectiveness of CSU treatment and disease control through therapy. It provides information on how well a given therapy controls urticaria over the last four weeks [15]. The questionnaire consists of four questions: frequency of urticaria symptoms (wheals/angioedema and itching), impact on QoL, therapy effectiveness, and evaluation of whether the disease was adequately controlled by therapy over the last month. The questions relate to the frequency of wheals/angioedema and itching, the impact of CSU on QoL, and the patient’s opinion on disease control. Responses are scored from 0 to 4, with the total score ranging from 0 to 16. Higher scores indicate better disease control [15]. Patients also completed the standard dermatological questionnaire, the DLQI, which includes ten questions about the impact of the disease on daily life, as well as on work duties, hobbies, and other leisure activities, and the impact on relationships with others and sleep quality over the past week [1]. DLQI values are calculated by summing the points for each question, where the response “very much” is worth three points, “a lot” two points, “a little” one point, and “not at all” zero points. The total DLQI score can range from 0 to 30 points (the higher the DLQI score, the more the patient’s QoL is impaired).

Patients also completed the PSQI questionnaire, which assesses their usual sleep habits over the past month and serves to evaluate sleep quality and sleep disturbances [16]. The PSQI analyzes nineteen items divided into seven “component” scores that assess subjective sleep quality, sleep duration, sleep disturbances, sleep latency, habitual sleep efficiency, use of sleep medication, and daytime dysfunction [16]. PSQI responses are scored from zero to three, with zero indicating no difficulty and three indicating severe difficulty. The total score can range from 0 to 21, and a PSQI score > 5 indicates a significant sleep disorder.

#### 2.2.2. Saliva Sampling and Determination of Salivary Melatonin by ELISA

Each participant received instructions on the proper collection of saliva samples, which was to be done in the evening between 10:00 p.m. and 12:00 a.m. Saliva sampling and questionnaire completion during the examination were carried out both during visits to the clinic and at home. Each participant was given guidelines on the correct procedure for collecting saliva samples, which included rules on food, drink, and alcohol consumption; smoking; and proper oral hygiene before saliva collection. Saliva collection was conducted in the evening in a dark room, at the recommended time between 10:00 p.m. and 12:00 a.m. The saliva collection method was performed using an indirect method with commercial FALCON saliva collection tubes (Sarstedt, Nümbrecht, Germany) [17,18].

The collected saliva samples were first stored (for up to three days) in a refrigerator (at a temperature of 4 to 8 °C) and then in a freezer (at −80 °C) until ELISA testing was performed. ELISA determined melatonin levels in saliva at the Department of Clinical Chemistry of the aforementioned hospital. After thawing the saliva samples, they were centrifuged (for ten minutes at 2000–3000 rpm) to remove any visible food debris. The minimum required volume of saliva samples directly pipetted into the wells was 0.5 mL. With proper sample preparation and buffer solution dilution, as specified in the melatonin kit, and adherence to protocol, the measurement of salivary melatonin in patients with CSU was enabled. Appropriate reagents (BÜHLMANN Laboratories AG, Schönenbuch, Switzerland) were applied, following the strict instructions of the manufacturer. The ELISA kit for salivary melatonin uses Kennaway G280 antibodies, with prior sample treatment by sodium hydroxide, followed by neutralization with hydrochloric acid (Direct Saliva MELATONIN—BUHLMANN Diagnostics. Available from: https://www.buhlmannlabs.ch/, accessed on 15 February 2020). Third-generation commercial tests have very high sensitivity (on the order of 1 pg/mL) for ELISA in plasma and saliva. The values of salivary melatonin were recorded as either low or standard, with a low melatonin value considered less than 4.2 pg/mL. The lower limit of analytical sensitivity of the assay was chosen as 0.3 pg/mL, as recommended by the manufacturer. The samples were stored until analysis at −20 degrees. According to the test manufacturer, samples are stable at this temperature for more than 6 months. Our samples were stored until analysis for 1–6 months.

#### 2.2.3. Statistical Methods

For salivary melatonin, SQ, and QoL, the normality of distribution for both study groups was determined by the Kolmogorov–Smirnov test. The results exhibited a non-normal distribution, so we used the Mann–Whitney test to compare the values between the groups. To calculate effect size, we used r = Z/√N. Fisher’s exact test was used for comparing frequencies, with the effect size quantified as Cramér’s V. The following criteria were used for interpretation: 0.14–0.36 = small effect size, 0.36–0.50 = moderate, and >0.5 = large effect size. The relationship between variables was analyzed using Spearman’s correlation. The following criteria were applied for interpretation: r > 0.9 indicates a robust correlation, 0.7–0.9 strong, 0.5–0.7 moderate, and 0.25–0.5 weak. Values of r > 0.250 with *p* < 0.05 were interpreted. We calculated odds ratios (ORs) with corresponding 95% confidence intervals (CIs) for categorical variables. For the interpretation of odds ratios, OR = 1.5 was considered mild, >3 moderate, and >9 large. The mediation model tested the mediating role of sleep quality in the relationship between dermatologic QoL and salivary melatonin levels, controlling for the effect of age and gender. The analysis was conducted using the PROCESS macro v4.3 with 5000 bootstrap samples and 95% confidence intervals. The commercial software IBM SPSS 22 was used (IBM Corp, Armonk, NY, USA).

## 3. Results

### 3.1. Salivary Melatonin Levels in CSU Patients and Comparison with Healthy Subjects (Control Group)

**Figure 1:**
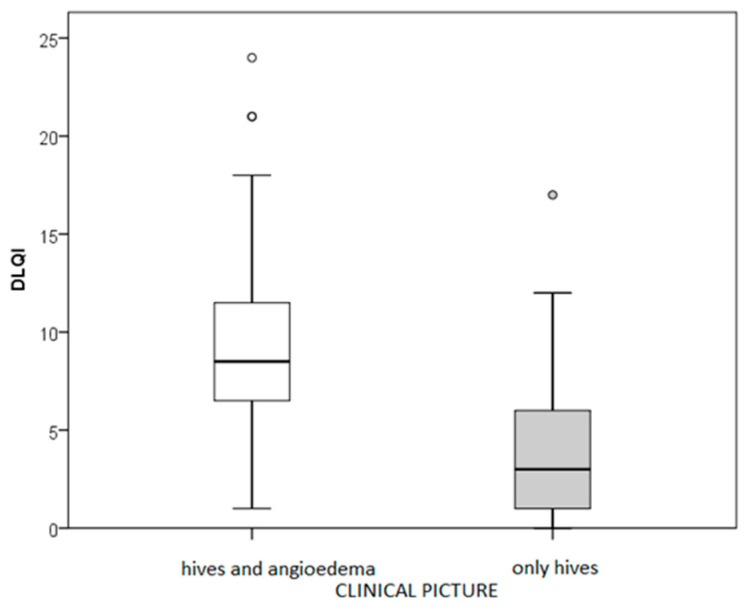
Urticaria activity effects on quality of life (DLQI). Circles present outliers.

The inter-assay coefficient of variation for salivary melatonin was 1.26. The median value of salivary melatonin in CSU patients was statistically significantly lower than that in healthy individuals, with a large effect size (0.2 vs. 15.9 pg/mL; *p* < 0.001; r = 0.669 Figure 1). Low salivary melatonin levels (<0.42 pg/mL) were found much more frequently in CSU patients (34/38 patients; 90%) compared to healthy subjects (7/38 healthy individuals; 18%) (*p* < 0.001, V = 0.713; Table 1). In the unadjusted analysis, CSU was included as the independent variable and showed that individuals with lower melatonin levels were significantly more likely to have CSU compared with those with higher melatonin levels (OR = 37.6; 95% CI 10.0–141.1).

The melatonin level did not depend on age in the whole sample (Table 2). Only correlations with *r* > 0.25 and *p* < 0.05 are considered significant.

Additionally, the prevalence of low melatonin levels did not differ between genders.

Looking at the relationship between melatonin levels (total sample) and the QoL of CSU patients, melatonin values were moderately negatively correlated with the DLQI (impaired QoL) (*r* = −0.606; *p* < 0.001) and PSQI (sleep disturbances) (*r* = −0.536; *p* < 0.001; Table 2). As impaired dermatologic QoL and impaired sleep quality increase, melatonin levels decrease (better QoL and sleep are associated with higher salivary melatonin levels). The melatonin level was not related to QoL or sleep quality in CSU patients (Table 3). It was related to sleep quality and age in the control group (*r* = −0.586 and *r* = −0.354; *p* ≤ 0.025).

### 3.2. Association of CSU Activity with Sleep Quality and Quality of Life in Patients

The activity of CSU (UAS7) was strongly and positively linearly related to impaired QoL (DLQI) and weakly positively related to sleep quality (*r* = 0.762 and 0.351; *p* ≤ 0.031). As disease activity increases, both impaired QoL and sleep quality worsen. Additionally, disease activity (UAS7) negatively correlated with disease control (UCT; *r* = −0.438; *p* = 0.006) and positively with itch intensity and the number of hives (*r* = 0.943 and 0.889; *p* < 0.001). However, disease activity (UAS7) did not correlate with the duration of CSU or the patient’s age.

In total, 47% of patients had urticaria alone, and the rest had urticaria combined with angioedema. People with a combination of urticaria and angioedema had a more impaired QoL than people with only urticaria (*p* = 0.004; *r* = −0.471; Figure 1).

Disease control (UCT) weakly negatively linearly correlated with impaired QoL (*r* = −0.454; *p* = 0.004) but did not correlate with sleep quality. As CSU control improves, impaired QoL in patients decreases. UCT also negatively correlated with UAS7, itch intensity, and the number of hives but did not correlate with disease duration or age. The disease duration did not correlate linearly with patient QoL, sleep, or melatonin concentration. When the disease duration was dichotomized with a 12-month threshold, there were no significant differences in QoL, sleep quality, age, or UCT and UAS7 values.

**Figure 2:**
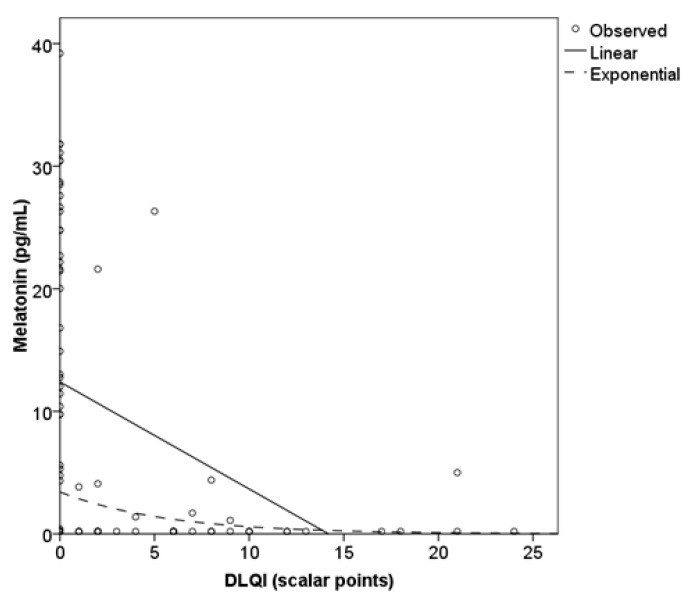
Relationship between Dermatology Life Quality Index (DLQI) and salivary melatonin.

Impaired QoL in patients correlated more strongly with itch intensity (*r* = 0.740) than with the number of hives *(r* = 0.646). Impaired sleep quality significantly correlated with itch intensity (*r* = 0.364) but did not correlate with the number of hives. The concentration of salivary melatonin did not correlate with either itch or hives. A scatter plot shows that the relationship between melatonin and the DLQI somewhat better explains an exponential equation (*y* = 3.4 × exp(−0.2 × *x*); *R*^2^ = 0.218) than a linear one (*y* = 12.4 − 0.9*x*; *R*^2^ = 0.188; Figure 2).

### 3.3. Association of Salivary Melatonin Concentration with Sleep Quality and Quality of Life

In the CSU patients, salivary melatonin did not correlate with age, dermatologic QoL, sleep quality, disease duration, UAS7 (dis act), the number of hives, itch intensity, or UCT (dis control)**.**

**Figure 3:**
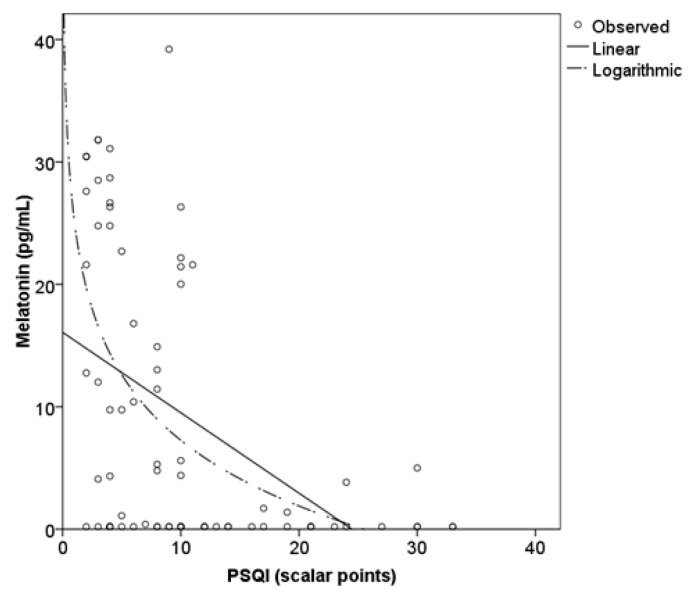
Relationship between sleep quality and salivary melatonin.

Additionally, to represent the relationship between melatonin levels and PSQI (sleep quality) in the total sample, the scatter plot suggests that a logarithmic equation (*y* = 25.1 − 7.8 × ln(*x*); *R*^2^ = 0.279; Figure 3) describes the relationship somewhat better than a linear one (*y* = 16.1 − 0.7*x*; *R*^2^ = 0.219).

The mediation analysis on the whole sample (CSU and controls) examined the PSQI as a mediator of the association between the DLQI and salivary melatonin, controlling for age and gender. The total effect of the DLQI on melatonin was significant (unstandardized coefficient B = −0.803; standard error (SE) = 0.214; *p* = 0.004). After including the PSQI in the model, the direct effect of the DLQI on melatonin was reduced, and the CI included 0 (B = −0.455; SE = 0.271; 95% CI −0.996 to 0.084), while the indirect effect through the PSQI was statistically significant (B = −0.347; 95% CI = −0.679 to −0.080), indicating partial mediation. Age and gender did not have a significant influence. Since the relationship was better modeled by an exponential and logarithmic relationship, mediation analysis was also conducted on log-transformed data, and the mediation effect of the PSQI was also found (B = −0.028; 95% CI = −0.059 to −0.006).

## 4. Discussion

Compared to healthy individuals, our CSU patients generally had lower melatonin levels, indicating impaired sleep quality, which particularly reflects the negative impact of itching, the most common subjective symptom of urticaria which disrupts their QoL [6,19,20]. Itching is one of the most significant factors that, in addition to its negative impact on QoL, adversely affects sleep quality and interferes with daily activities [20,21]. Our CSU patients usually find itching more difficult to tolerate than the appearance of wheals, with itching having a significantly more significant impact on their QoL than the wheals. Sleep disturbances are frequent in patients with chronic dermatoses, particularly those accompanied by itching or pain (atopic dermatitis, urticaria, prurigo, acne vulgaris, psoriasis, lichen planus, etc.) [22]. Recurrent/chronic nocturnal itching (chronic) is a significant problem in these patients, often leading to disrupted sleep. According to one study, sleep disturbances are twice as common in CSU patients compared to healthy individuals and are associated with CSU activity/UAS7 [23]. Their common sleep disturbances (on average, three nights per week) are often inadequately managed/resolved (in 48% of patients) [24]. Compared to other dermatoses (such as psoriasis), more CSU patients reported sleep difficulties than psoriasis patients [25]. So, CSU activity impacts sleep, while patients with more severe CSU had poorer sleep quality and lower QoL. In our patients, a more substantial impairment in sleep quality and dermatologic QoL was linked to lower melatonin levels, indicating that better QoL and sleep were associated with higher melatonin levels. Sleep quality and dermatologic QoL were conceptualized in this research as mediators linking CSU-related symptom burden with circadian dysregulation and reduced melatonin secretion, while age and gender were considered confounders. Sleep quality emerged as a mediator of the relationship between QoL and melatonin in the whole sample (CSU and healthy subjects). In CSU patients, melatonin concentration was not associated with age, dermatologic QoL, sleep quality, disease duration, CSU severity (hives, itching), or disease control, which suggests that melatonin may be more of a non-specific marker of sleep disturbance than a CSU severity. According to one study, CSU patients had more sleepiness and a greater tendency toward daytime sleepiness and their QoL positively correlated with sleep quality while negatively with CSU duration, the apnea–hypopnea index, apnea duration, total number of respiratory events, and the number of apneas [23]. Ultimately, sleep deprivation and the loss of sleep negatively impact work performance and daily functioning, significantly affecting patients’ social life, and are associated with a shorter lifespan [24].

In CSU patients, the disease significantly impacts their QoL due to the frequent hives/itching, the chronic disease nature, the frequency of relapses, and resistance to existing therapies [1,22,23,24,25,26]. In our patients, CSU most often moderately impacted QoL (36.8% experienced a moderate impact; 21% experienced a very significant or highly significant impact), which corresponds with literature data. The only factor related to their QoL was the activity of the clinical presentation (mostly itch). Also, for CSU patients’ QoL, the level of disease control is very important (the UCT score). Thus, in our patients, disease control was significantly related to QoL, so better urticaria control was associated with their better QoL. We also observed the impact of age on CSU control, i.e., our patients who had well-controlled CSU were older than those with poor disease control, which suggests that older individuals are more attentive to disease management and avoiding triggering factors (if they exist). According to one Japanese study, the CSU activity strongly positively correlates with their impaired QoL, while disease control/UCT strongly inversely correlated with impaired QoL [27]. Also, disease control/UCT weakly and negatively correlates with QoL but does not correlate with CSU duration or age, suggesting that as disease control improves, the impairment in QoL decreases [27]. So, the use of the UCT questionnaire is recommended due to its practicality and sensitivity to treatment outcomes, which was confirmed in our study, as evidenced by the confirmed a link between UAS, UCT, and DLQI scores. Additionally, CSU control/UCT inversely correlated with CSU activity/UAS7 (itch and hives), i.e., as disease control improves, CSU activity decreases [28]. According to the literature, generally, poorer QoL does not depend on age, gender, or disease duration, which aligns with our findings [29,30,31]. Additionally, one study on CSU patients found a significant correlation between fatigue and QoL. The presence of accompanying angioedema is also important, as our CSU patients with concurrent angioedema experienced impaired QoL, poorer disease control, and a more severe CSU form compared to those with wheals alone, without angioedema. According to one broader study, angioedema appeared in 66% of patients and significantly impacted their QoL, which aligns with our findings [32]. According to the international prospective AWARE study results, the factors most affecting their QoL were the frequent wheals/angioedema occurrence, intense itching, disrupted sleep, fatigue, and reduced concentration [33]. All this suggests that multiple factors influence their QoL.

Recurrent hives, itch and impaired sleep often lead to psychological disturbances such as irritability, nervousness, anxiety, or heightened stress perception. CSU’s chronic nature, the inability to identify the cause/trigger for CSU, and the frequent lack of response to treatment further contribute to these issues [33,34,35]. However, this impact is highly subjective; for instance, patients with a mild CSU presentation may experience significantly impaired QoL, while those with a more severe form of CSU may not necessarily have a significantly impaired QoL [33]. During patient treatment and follow-up, a decreased DLQI score (increased QoL) and disease activity/UAS7 was observed [33]. In the real life of CSU patients, intense itching and hives are unpredictable and often impact sleep disturbances, as well as daily life, work, school/university, or sports activities. The disease interferes with their daily routines and sleep and consequently affects their psychological state. So, urticaria is among the dermatologic diseases with the highest prevalence of associated psychiatric disorders [36]. If anxiety disorders or depressive episodes are present (which occur in 30% of CSU patients), they further contribute to the impaired QoL of these patients [37].

When examining sleep disorders generally and the potential melatonin therapeutic use for patients (e.g., those with sleep disorders), experimental studies have shown its successful therapeutic use for inflammatory neurological and gastrointestinal conditions as well. Furthermore, international expert opinions and recommendations suggest the possible treatment of insomnia through systematic melatonin use to mimic the physiological melatonin secretion cycle. Clinically, melatonin is recommended for patients with sleep disorders and psychiatric conditions as a potential pharmacotherapeutic option [38,39]. The current treatment of CU (according to global guidelines) includes second-generation H1 antihistamines (up to four tablets per day) or, if necessary, short courses of systemic corticosteroids, omalizumab, or cyclosporine, as well as dupilumab and remibrutinib [1,40]. Drugs recently or currently in clinical trials target multiple pathways/molecules (e.g., intracellular signaling molecules or receptors that regulate inflammatory cell chemotaxis: Siglec-8 that influences eosinophil apoptosis and mast cell mediators and other factors): rilzabrutinib, barzovolimab, briquilimab, povorcitinib, etc. [40]. Also, the confirmed low melatonin levels in CSU patients may have clinical relevance, including its potential therapeutic use for supplementation, to improve sleep and potentially reduce symptoms. Hypothetically, melatonin could potentially be used as a therapy for immunomodulation (due to its antioxidant and immunomodulation/anti-inflammatory properties), light therapy (using controlled light exposure to regulate the body’s natural melatonin production and circadian rhythms), or targeting inflammation pathways (potentially mimicking the effects of melatonin on its MT receptors or pathways involved in its antioxidant/immunomodulatory actions). Also, melatonin could stabilize mast cell membranes and inhibit degranulation, which could be a useful tool for treating diseases characterized by acute/chronic inflammatory processes involving mast cells (e.g., urticaria). So, there is a clear need for further studies exploring potential therapeutic strategies to inhibit their activation.

However, the limitations of this research are: (1) its cross-sectional design; (2) one-time, single-night melatonin measurement (while CSU activity was tracked over seven days, which may impact interpretation); (3) the relative low number of participants; (4) some other factors which may influence sleep and cause poor sleep were not be measured/analyzed, and (5) the use of the ELISA method (e.g., a method like MS/MS could be more suitable). As for individual melatonin measurements, this may affect interpretation, as potentially more measurements would provide a better insight into melatonin values. It may also be mentioned that salivary melatonin levels are low and that samples must be collected in low light (to prevent instantaneous degradation and suppression), which could make field studies more difficult. In addition, samples can potentially be contaminated with food particles or blood, so careful and supervised collection is necessary to avoid invalid results. However, using saliva for melatonin analysis has many advantages, as serum melatonin analysis requires medical personnel, is potentially stressful, and is not practical for repeated overnight sampling. Saliva is safer and easier to collect and provides similar accuracy for the onset of melatonin action in low light. While urine melatonin analysis involves measuring the metabolite 6-sulfatoxymelatonin (representing cumulative production over several hours), saliva provides a direct, “real-time” measurement of current plasma levels, making it more suitable for determining the exact timing of circadian rhythms (phases). Regarding our methodology, several circadian and sleep studies have defined low nocturnal or basal salivary melatonin levels using cutoff values in the approximate range of 3–5 pg/mL, supporting the biological relevance of our chosen cutoff value. Representative studies using similar or alternative cutoff values can be cited, while acknowledging the variability in cutoff selection across studies, which further increases transparency, places our biomarker definition within the existing literature and helps interpret the clinical and biological significance of low melatonin levels in the context of CSU [41,42,43,44].

So, based on previous articles and research, further clinical studies on large samples of patients should be expected and welcomed to reveal the etiology of the low melatonin level in CSU patients and evaluate its therapeutic potential [6,19,41].

## 5. Conclusions

In CSU patients, itching impairs sleep, which consequently reduces the melatonin level (measured in saliva) and may impair QoL or cause psychological/psychiatric disorders. Our data suggest that melatonin could be a more non-specific marker of sleep disturbance rather than of CSU severity. Sleep quality may act as a mediator linking dermatology-related QoL with circadian dysregulation and reduced melatonin secretion. These results highlight the importance of appropriate and high-quality treatment for these patients, taking into account the various CSU aspects and daily activities and life, including sleep, which accounts for one-third of life. A multidisciplinary approach to the treatment of CSU is needed, which may include collaboration between dermatologists, clinical psychologists, or psychiatrists, when appropriate, as well as efforts to develop new therapeutic options for them.

## Figures and Tables

**Table 1:** Prevalence of low and normal melatonin levels in groups.

Group	Low Melatonin (<0.42 pg/mL)	Normal Melatonin (>0.42 pg/mL)	Sum	*p*	V
CSU	34	4	38		
Healthy	7	31	38		
sum	41	35	76	0.001	0.713

**Table 2:** Spearman’s correlations (N = 76)—total sample.

Variable		Melatonin	Age	DLQI	PSQI
Melatonin	*r*	1	−0.231	−0.606	−0.536
	*p*		0.045	<0.001	<0.001
Age	*r*	−0.231	1	0.113	0.295
	*p*	0.045		0.329	0.010
Dermatology Life Quality Index	*r*	−0.606	0.113	1	0.535
	*p*	<0.001	0.329		<0.001
Pittsburgh Sleep Quality Index	*r*	−0.536	0.295	0.535	1
	*p*	<0.001	0.010	<0.001	

**Table 3:** Spearman’s correlations (N = 38)—CSU group.

Variable		Melatonin	Age	DLQI	PSQI
Melatonin	*r*	1	−0.130	−0.075	0.004
	*p*		0.435	0.655	0.981
Age	*r*	−0.130	1	0.267	0.370
	*p*	0.435		0.105	0.022
Dermatology Life Quality Index	*r*	−0.075	0.267	1	0.453
	*p*	0.655	0.105		<0.004
Pittsburgh Sleep Quality Index	*r*	0.004	0.370	0.453	1
	*p*	0.981	0.022	0.004	

## Data Availability

The original contributions presented in this study are included in the article. Further inquiries can be directed to the corresponding author.

## References

[1] Zuberbier T., Abdul Latiff A.H., Abuzakouk M., Aquilina S., Asero R., Baker D., Ballmer-Weber B., Bangert C., Ben-Shoshan M., Bernstein J.A. (2022). The international EAACI/GA^2^LEN/EuroGuiDerm/APAAACI guideline for the definition, classification, diagnosis, and management of urticaria. Allergy.

[2] Türk M., Kocatürk E., Ertaş R., Ensina L.F., Mariel Ferrucci S., Grattan C., Vestergaard C., Zuberbier T., Maurer M., Giménez-Arnau A.M. (2024). A global perspective on stepping down chronic spontaneous urticaria treatment: Results of the Urticaria Centers of Reference and Excellence SDown-CSU study. Clin. Transl. Allergy.

[3] Kaplan A., Lebwohl M., Giménez-Arnau A.M., Hide M., Armstrong A.W., Maurer M. (2023). Chronic spontaneous urticaria: Focus on pathophysiology to unlock treatment advances. Allergy.

[4] Zhou B., Li J., Liu R., Zhu L., Peng C. (2022). The role of crosstalk of immune cells in pathogenesis of chronic spontaneous urticaria. Front. Immunol..

[5] Amaral F.G.D., Cipolla-Neto J. (2018). A brief review about melatonin, a pineal hormone. Arch. Endocrinol. Metab..

[6] Bešlić I., Vrtarić A., Bešlić A., Škrinjar I., Crnković D., Lugović-Mihić L. (2023). Salivary melatonin values significantly correlate with reduced quality of life in chronic spontaneous urticaria patients: A pilot study. Acta Clin. Croat..

[7] Gómez-Moreno G., Guardia J., Ferrera M.J., Cutando A., Reiter R.J. (2010). Melatonin in diseases of the oral cavity. Oral Dis..

[8] Hardeland R. (2017). Taxon- and site-specific melatonin catabolism. Molecules.

[9] Laakso M.L., Porkka-Heiskanen T., Alila A., Stenberg D., Johansson G. (1990). Correlation between salivary and serum melatonin: Dependence on serum melatonin levels. J. Pineal Res..

[10] Carrascal L., Nunez-Abades P., Ayala A., Cano M. (2018). Role of Melatonin in the Inflammatory Process and Its Therapeutic Potential. Curr. Pharm. Des..

[11] Kuna M., Štefanović M., Ladika Davidović B., Mandušić N., Birkić Belanović I., Lugović-Mihić L. (2023). Chronic urticaria biomarkers IL-6, ESR and CRP in correlation with disease severity and patient quality of life-a pilot study. Biomedicines.

[12] Antia C., Baquerizo K., Korman A., Bernstein J.A., Alikhan A. (2018). Urticaria: A comprehensive review: Epidemiology, diagnosis, and work-up. J. Am. Acad. Dermatol..

[13] Młynek A., Zalewska-Janowska A., Martus P., Staubach P., Zuberbier T., Maurer M. (2008). How to assess disease activity in patients with chronic urticaria?. Allergy.

[14] Hawro T., Ohanyan T., Schoepke N., Metz M., Peveling-Oberhag A., Staubach P., Maurer M., Weller K. (2018). The Urticaria Activity Score-validity, reliability, and responsiveness. J. Allergy Clin. Immunol. Pract..

[15] Weller K., Groffik A., Church M.K., Hawro T., Krause K., Metz M., Martus P., Casale T.B., Staubach P., Maurer M. (2014). Development and validation of the Urticaria Control Test: A patient-reported outcome instrument for assessing urticaria control. J. Allergy Clin. Immunol..

[16] Buysse D.J., Reynolds C.F., Monk T.H., Berman S.R., Kupfer D.J. (1989). The Pittsburgh Sleep Quality Index: A New Instrument for Psychiatric Practice and Research. Psychiatry Res..

[17] Meštrović-Štefekov J., Lugović-Mihić L., Hanžek M., Bešlić I., Japundžić I., Karlović D. (2022). Salivary Cortisol values and personality features of atopic dermatitis patients: A prospective study. Dermatitis.

[18] Ćesić D., Lugović-Mihić L., Ferček I., Grginić A.G., Jelić M., Bešlić I., Tambić Andrašević A. (2021). Salivary microbiota is significantly less diverse in patients with chronic spontaneous urticaria compared to healthy controls: Preliminary results. Life.

[19] Can A., Tuzer O.C. (2023). The evaluation of melatonin levels in chronic spontaneous urticaria: A case control study. Allergy Asthma Proc..

[20] Abdel Latif O.M. (2017). Impact of severity of CSU on sleep, anxiety and depressive symptoms in adults. Eur. Acad. Res..

[21] Verhoeven E.W., de Klerk S., Kraaimaat F.W., van de Kerkhof P.C., de Jong E.M., Evers A.W. (2008). Biopsychosocial mechanisms of chronic itch in patients with skin diseases: A review. Acta Derm. Venereol..

[22] Kaaz K., Szepietowski J.C., Matusiak Ł. (2019). Sleep quality among adult patients with chronic dermatoses. Postepy Dermatol. Allergol..

[23] Ates H., Firat S., Buhari G.K., Keren M., Cifci B., Erkekol F.Ö. (2022). Relationships between quality of life, sleep problems, and sleep quality in patients with chronic idiopathic urticaria. J. Cosmet. Dermatol..

[24] Giménez-Arnau A., Maurer M., Bernstein J., Staubach P., Barbier N., Hua E., Severin T., Joubert Y., Janocha R., Balp M.M. (2022). Ligelizumab improves sleep interference and disease burden in patients with chronic spontaneous urticaria. Clin. Transl. Allergy.

[25] Gupta M.A., Gupta A.K., Schork N.J., Ellis C.N. (1994). Depression modulates pruritus perception: A study of pruritus in psoriasis, atopic dermatitis, and chronic idiopathic urticaria. Psychosom. Med..

[26] Filiz S., Kutluk M.G., Uygun D.F.K. (2019). Headache deteriorates the quality of life in children with chronic spontaneous urticaria. Allergol. Immunopathol..

[27] Nakatani S., Oda Y., Washio K., Fukunaga A., Nishigori C. (2019). The Urticaria Control Test and Urticaria Activity Score correlate with quality of life in adult Japanese patients with chronic spontaneous urticaria. Allergol. Int..

[28] Kocatürk E., Kızıltaç U., Can P., Öztaş Kara R., Erdem T., Kızıltaç K., Sahillioğlu N., Gelincik A., Maurer M., Weller K. (2019). Validation of the Turkish version of the Urticaria Control Test: Correlation with other tools and comparison between spontaneous and inducible chronic urticaria. World Allergy Organ. J..

[29] Sánchez-Borges M., Ansotegui I.J., Baiardini I., Bernstein J., Canonica G.W., Ebisawa M., Gomez M., Gonzalez-Diaz S.N., Martin B., Morais-Almeida M. (2021). The challenges of chronic urticaria part 1: Epidemiology, immunopathogenesis, comorbidities, quality of life, and management. World Allergy Organ. J..

[30] Paudel S., Parajuli N., Sharma R.P., Dahal S., Paudel S. (2020). Chronic urticaria and its impact on the quality of life of nepalese patients. Dermatol. Res. Pract..

[31] Ertaş Ş.K., Ertaş R. (2021). Fatigue is common and predicted by female gender and sleep disturbance in patients with chronic spontaneous urticaria. J. Allergy Clin. Immunol. Pract..

[32] Maurer M., Abuzakouk M., Bérard F., Canonica W., Oude Elberink H., Giménez-Arnau A., Grattan C., Hollis K., Knulst A., Lacour J.P. (2017). The burden of chronic spontaneous urticaria is substantial: Real-world evidence from ASSURE-CSU. Allergy.

[33] Maurer M., Giménez-Arnau A., Ensina L.F., Chu C.Y., Jaumont X., Tassinari P. (2020). Chronic urticaria treatment patterns and changes in quality of life: AWARE study 2-year results. World Allergy Organ. J..

[34] Mosnaim G., Patil D., Kuruvilla M., Hetherington J., Keal A., Mehlis S. (2024). Patient and physician perspectives on disease burden in chronic spontaneous urticaria: A real-world US survey. Ann. Allergy Asthma Immunol..

[35] Weller K., Winders T., McCarthy J., Raftery T., Saraswat P., Constantinescu C., Balp M.M., Bernstein J.A. (2025). Urticaria voices: Real-world experience of patients living with chronic spontaneous urticaria. Dermatol. Ther..

[36] Gonçalo M., Gimenéz-Arnau A., Al-Ahmad M., Ben-Shoshan M., Bernstein J.A., Ensina L.F., Fomina D., Galvàn C.A., Godse K., Grattan C. (2021). The global burden of chronic urticaria for the patient and society. Br. J. Dermatol..

[37] Konstantinou G.N., Konstantinou G.N. (2019). Psychiatric comorbidity in chronic urticaria patients: A systematic review and meta-analysis. Clin. Transl. Allergy.

[38] Neubauer D.N. Pharmacotherapy for Insomnia in Adults. https://www.uptodate.com/contents/pharmacotherapy-for-insomnia-in-adults.

[39] Bešlić I., Lugović-Mihić L., Vrtarić A., Bešlić A., Škrinjar I., Hanžek M., Crnković D., Artuković M. (2023). Melatonin in Dermatologic Allergic Diseases and Other Skin Conditions: Current Trends and Reports. Int. J. Mol. Sci..

[40] Kolkhir P., Fok J.S., Kocatürk E., Li P.H., Okas T.L., Marcelino J., Metz M. (2025). Update on the Treatment of Chronic Spontaneous Urticaria. Drugs.

[41] Ungurianu A., Marina V. (2025). Melatonin and Cortisol Suppression and Circadian Rhythm Disruption in Burnout Among Healthcare Professionals: A Systematic Review. Clin. Pract..

[42] Pullman R.E., Roepke S.E., Duffy J.F. (2012). Laboratory validation of an in-home method for assessing circadian phase using dim light melatonin onset (DLMO). Sleep Med..

[43] Skubic C., Zevnik U., Nahtigal K., Dolenc Grošelj L., Rozman D. (2025). Circadian Biomarkers in Humans: Methodological Insights into the Detection of Melatonin and Cortisol. Biomolecules.

[44] Ambaldhage V., Naik Purnachandrarao N., Alaparthi R.K., Yelamanchili S. (2016). Chemical of darkness (Melatonin): A ray of glow to dentistry. J. Indian Acad. Oral Med. Radiol..

